# Food variety consumption and household food insecurity coping strategies after the 2010 landslide disaster – the case of Uganda

**DOI:** 10.1017/S1368980016001397

**Published:** 2016-06-09

**Authors:** Peter M Rukundo, Arne Oshaug, Bård A Andreassen, Joyce Kikafunda, Byaruhanga Rukooko, Per O Iversen

**Affiliations:** 1 Department of Human Nutrition and Home Economics, Kyambogo University, Kampala, Uganda; 2 Department of Nutrition, Institute of Basic Medical Sciences, University of Oslo, 1046 Blindern, 0317 Oslo, Norway; 3 Faculty of Applied Health Sciences, Oslo and Akershus University College of Applied Sciences, Oslo, Norway; 4 Norwegian Centre for Human Rights, Faculty of Law, University of Oslo, Oslo, Norway; 5 School of Food Technology, Nutrition and Bio-engineering, Makerere University, Makerere, Uganda; 6 School of Liberal and Performing Arts, Makerere University, Makerere, Uganda; 7 Department of Haematology, Oslo University Hospital, Oslo, Norway

**Keywords:** Disaster, Food insecurity, Nutrition, Resettlement

## Abstract

**Objective:**

To evaluate the nutritional situation of the victims of the 2010 landslide disaster in Uganda, food varieties consumed and coping strategies were assessed.

**Design:**

Cross-sectional. Food variety scores (FVS) were obtained as the total of food items eaten over the last week while an index was based on severity weighting of household food insecurity coping strategies. We included 545 affected and 533 control households.

**Setting:**

Victims in the affected Bududa district in Eastern Uganda and those victims resettled in the Kiryandongo district, Western Uganda.

**Results:**

Adjusted for covariates, in Bududa significantly higher mean FVS were observed among: affected than controls; farmers than others; and relief food recipients. Control households scored higher means (se) on severity of coping: 28·6 (1·3) *v*. 19·2 (1·2; *P*<0·01). In Kiryandongo, significantly higher FVS were observed among: control households; household heads educated above primary school; those with assets that complement food source; and recipients of relief food. Severity of coping was significantly higher among affected households and non-recipients of relief food. Affected households had a higher likelihood to skip a day without eating a household meal in Bududa (OR=2·31; 95 % CI 1·62, 3·29; *P*<0·01) and Kiryandongo (OR=1·77; 95 % CI 1·23, 2·57; *P*<0·01).

**Conclusions:**

Whereas FVS and severity of coping showed opposite trends in the two districts, resettlement into Kiryandongo led to severe coping experiences. Administrative measures that provide a combination of relief food, social protection and resettlement integration may offset undesirable coping strategies affecting diet.

The count of different food varieties used by a household, denoted as food variety score (FVS), is among the proxy indicators used to evaluate dietary quality and adequacy^(^ ^1^ ^)^. The notion of adequacy is particularly important in the description of food as a fundamental human right^(^ ^2^ ^)^. It is also vital in understanding the immediate determinants of a wide range of nutritional-health outcomes with consequences for survival, disease and mortality^(^ ^3^ ^–^ ^5^ ^)^. Achieving an adequate diet can be complex. It largely encompasses the availability and accessibility to food that is sufficient in quality and quantity to satisfy the dietary needs of individuals, free from adverse substances, and acceptable within a given culture^(^ ^2^ ^)^.

In assessing diet, understanding how households cope with situations where there is inadequate food, or a lack of means for its procurement, may facilitate the process of addressing the underlying determinants associated with the type, quality and quantity of food and diet that are available and accessible to a given population^(^ ^6^ ^–^ ^9^ ^)^. These coping strategies, unlike long-term and permanent adaptive strategies, are considered as temporary fall-back mechanisms and adjustments in ways of life by households so as to deal with a short-term insufficiency of food^(^ ^10^ ^)^. However, the situation of coping can be misinterpreted particularly when there are seasonal changes and disruptive events like disasters, which expose households to varying realities of inequality that affect access to adequate food, thus compelling some to ration the quality, quantity and variety of food consumed^(^ ^11^ ^,^ ^12^ ^)^.

In Uganda, the absence of dietary guidelines and reliable food composition data poses a limitation in the implementation of programmes on food and nutrition security, monitoring and early warning of looming food shortages. Natural disasters are also rampant and cyclic in some areas, thereby increasing the risk of vulnerability in this country where undernourishment already affects about one of every five people^(^ ^13^ ^)^. With an estimated 200 000 people in Uganda affected by natural disasters annually^(^ ^14^ ^)^, the problem has become acute and is cited as a constraint to the country’s development^(^ ^15^ ^)^. A particular case was a major landslide, considered the worst in the country’s history, which struck in the Bududa district of Eastern Uganda in March 2010. It claimed about 350 lives^(^ ^16^ ^,^ ^17^ ^)^ and affected another 10 000; about 10 % of whom were resettled over 300 km away in the Western Uganda district of Kiryandongo^(^ ^18^ ^)^.

The aim of the present study was to assess the food varieties consumed and household food insecurity coping strategies after the 2010 landslide disaster event. We surveyed two districts to compare two independent groups: affected households and controls. In doing so, the association of household sociodemographic variables with food variety and food insecurity coping strategies was investigated to establish the extent of variation. Given that no similar studies had been performed among households affected by such type of disaster, our study may inform follow-up actions and studies related to food and nutrition security in the study areas.

## Methods

### Study design

The present study was part of a cross-sectional research project that analysed food as a human right during disaster in Uganda^(^ ^19^ ^)^, explored perceptions on the right to adequate food in the aftermath of the 2010 landslide disaster in Eastern Uganda^(^ ^20^ ^)^, and assessed household food insecurity and diet diversity in the aftermath of that disaster^(^ ^21^ ^)^. Following the pre-survey site familiarization visits, sensitization meetings were held with district authorities. Subsequently, data collection assistants were recruited and trained. The survey pre-test was held between 12 August and 15 November 2012, while the data collection survey was performed from 19 November 2012 to 21 December 2012 to avoid possible bias during the Christmas and New Year festivities when most households often alter their usual dietary habits.

### Study population and participants

The study population was households in the two districts that hosted victims of the 2010 landslide disaster. Bududa district was chosen because its proneness to landslides^(^ ^16^ ^,^ ^22^ ^–^ ^24^ ^)^. In March 2010 its sub-county of Bukalasi was the site of one of the most devastating landslides in Uganda. More than 350 persons reportedly died and over 10 000 were affected^(^ ^16^ ^–^ ^18^ ^,^ ^25^ ^)^. In addition, Kiryandongo district was selected on the basis that it hosted nearly 1000 disaster-affected households who accepted the Government decision to be voluntarily resettled from Bukalasi into the Mutunda sub-county of Kiryandongo district in the aftermath of the landslide disaster. In order to establish the diet quality and household food insecurity coping situation of disaster victims, affected households were compared with controls in each district so as to estimate the extent of variation when the disaster and sociodemographic factors were taken into account.

In each district the affected group comprised the 2010 landslide disaster-affected households, while the controls were households from a randomly selected sub-county bordering the geographical area where the disaster-affected people were located. The controls were not selected from within the same homogeneous population of the affected households due to the ecological and complex nature of the disaster effect; to the extent that vital sub-county infrastructure like roads, a health centre and trading centre were destroyed, and several hundreds of deaths and displaced persons recorded. In addition, the two districts were examined independently in our study. As described in our publications elsewhere^(^ ^20^ ^,^ ^21^ ^)^, the two districts differed in demography, seasonality, climate, geography, traditional culture and tribe among others. Despite this non-homogeneity of the affected and control groups, we assumed that the situation of household food variety and food insecurity coping experiences were relatively the same in the affected and control areas prior to the landslide disaster event of 2010.

In computing sample size, we used the prevalence of undernourishment, a state of prolonged inability to acquire enough food^(^ ^26^ ^)^, as a proxy due to the absence of reliable effect measures of landslides on food insecurity and diet. The 19 % national estimate of undernourishment reported in the Uganda Nutrition Action Plan 2011–2016^(^ ^13^ ^)^ was therefore used to compute the sample size of control households and we assumed that the landslides had increased it to 29 % in the affected groups. Using a 1:1 ratio of affected to control groups, our computation used a significance level of 5 % and power of 80 % to yield a total sample size of 576 households per district. Based on the probability proportional to size precisions used in two recent surveys by the Uganda Bureau of Statistics^(^ ^27^ ^,^ ^28^ ^)^, we randomly targeted twelve households in a village; the smallest grouping of households from a defined enumeration area in Uganda. As adopted by Uganda’s Bureau of Statistics^(^ ^27^ ^,^ ^28^ ^)^ and Harvey and colleagues^(^ ^29^ ^)^, an extra twelve households was added to each group in each district to compensate for possible non-response. We therefore targeted 300 randomly selected households per sub-county with affected or controls, i.e. a total of 600 households per district and 1200 households in both districts.

Given the community and geographical organization of the study areas, a three-stage simple random sampling procedure was applied in each district. The first stage commenced with random selection of the control sub-county from a list of sub-counties neighbouring the already known sub-county with affected households, i.e. Bukalasi in Bududa district and Mutunda in Kiryandongo district. At the second stage, all villages and their corresponding estimates of number of households in each of the affected and control areas were listed and randomly assigned into twenty-five village units using probability proportional to size, hence a total of 100 villages in both districts. The third stage involved randomly selecting twelve households in each village from the household lists that were generated during the pre-survey mapping and listing exercise. Simple computer-generated random tables were used to obtain random numbers from a range of an ascending numbered list of village households. Households whose position on the list matched with the random numbers were identified and consulted for interviews.

### Interviews with the head of the household

The index respondent was the head of the household. Although we preferred to interview women respondents due to their role in food and nutrition security, the head of the household who was available and willing to participate was the one interviewed. The questionnaire structure emphasized closed-ended questions on sociodemographic characteristics, food insecurity coping strategies and the frequency of food intake. The recall period was 7 d prior to the interview date.

### Scoring the household food variety scores

The household’s FVS was computed as the number of different food items supposedly eaten by a household in the assigned recall period. As has been previously used in Uganda^(^ ^30^ ^,^ ^31^ ^)^, commonly eaten food varieties totalling seventy-two items were listed in twelve groups to facilitate a retrospective recall by the head of the household: (i) cereals and grains; (ii) legumes and pulses; (iii) starchy roots and tubers; (iv) vegetables; (v) fruits and fruit juices; (vi) poultry and eggs; (vii) meat and meat products; (viii) milk and milk products; (ix) fats and oils; (x) fish and fish products; (xi) refined sugar and confectioneries; and (xii) carbonated non-alcoholic beverages, condiments and spices. Using the information of food items eaten, we also computed FVS within each group to ascertain the number and types of food items that were consumed from each food group. The food group variety score facilitated the process to estimate, in relative terms, how households performed on the assigned food groups.

### Scoring the household coping strategies

A coping strategy score was generated based on the eleven strategies commonly used by households facing food insecurity threats in resource-limited settings, i.e. situations of inadequate food or a lack of means for its procurement. Given the emergency situation in the aftermath of the 2010 disaster and the absence of a gold standard for measuring food insecurity and related coping strategies, the study adapted a mix of experiences about food access, child hunger and food insecurity coping practices during emergencies, from the Household Food Insecurity Access Scale (HFIAS)^(^ ^32^ ^)^, the Community Childhood Hunger Identification Project (CCHIP) index^(^ ^33^ ^)^ and the Coping Strategy Index (CSI)^(^ ^6^ ^,^ ^7^ ^)^, respectively. The tools have been used in East and Southern Africa^(^ ^33^ ^–^ ^37^ ^)^. We specifically adapted three strategies from the HFIAS: on skipping meals, reducing portion sizes and reducing food for adults; five strategies from the CSI: on relying on less preferred and less expensive food, borrowing food, purchasing food on credit, seeking monetary support for food and children eating elsewhere due to no food; and two strategies from the CCHIP: on parents eating less food so children can eat and children eating less due to inadequate food or means for its procurement.

In each district, we recruited ten data collection assistants who were trained on the questionnaire content, interviewing and probing skills before pre-testing the survey tool. During the pre-test exercise, each coping strategy commonly deployed by households when faced by food insecurity challenges was adapted and ranked for severity using a scale of severity whose weights ranged from 1 to 4 points^(^ ^6^ ^)^. A weight of 4 points was assigned to what were perceived as the most serious coping strategies: skipping a day without eating a household meal (we considered three main household meals of breakfast, lunch and supper, while excluding snacks or other food eaten outside the household); children going to bed hungry; and allowing children to roam and eat elsewhere due to inadequate food in household. A weight of 3 points was assigned to: seeking financial credit to buy food; children eating less food; and borrowing food. A weight of 2 points was assigned to: limiting portion sizes at meals; reducing food for adults; eating less as a parent; and purchasing food on credit. The least weight of 1 point was assigned to relying on less preferred and less expensive foods. As such, the frequency of each coping strategy over the 7 d recall period was scored. In addition, the severity of coping to food insecurity was computed as a total of weighted scores.

A severity score for each coping strategy was computed by multiplying its weight value by the frequency of times a household reported as having experienced it over the last 7 d period^(^ ^6^ ^)^. For example, a single category 4 strategy experienced every day for the recall period of 7 d would have a maximum score of 28 points (4×7×1), while a category 1 strategy experienced every day would score 7 points (1×7×1). The total severity of coping score for each household was a total of the weighted scores for the eleven coping strategies. A maximum severity of coping score for a household that experienced all eleven strategies daily was 210 points ((4×7×3)+(3×7×3)+(2×7×4)+(1×7×1)). The analysed scores were computed into means.

### Statistical analyses

We used the statistical software package IBM SPSS Statistics Version 21 to report statistical associations and mean differences. Due to the existence of extreme values that affected normality of the data, crude mean differences in scores were tested using Levene’s independent-samples *t* test due to its suitability for application to both normally and non-normally distributed data. Given that the two dependent quantitative outcomes of food variety and weighted coping scores showed a moderate positive correlation (correlation coefficient *r*<0·9 in both districts), a one-way MANCOVA model was used to test for univariate and multivariate effects while controlling for the disaster effect and sociodemographic covariates: head of the household’s gender, age, level of education, household size, main source of livelihood, existence of assets relevant to food security and having received relief food. The model was suitable given that it also reports the adjusted univariate effect on each dependent outcome. Moreover, the violation of homogeneity of variance observed with FVS posed no threat to validity given that the Brown–Forsythe *F* and Welch’s *F* adjustments were significant when tested in a one-way independent ANOVA prior to performing MANCOVA.

Categorical variables with ‘yes’ or ‘no’ options for each coping strategy were analysed using the Pearson *χ* ^*2*^ odds ratio and are reported with their corresponding 95 % confidence interval and statistical significance of *P*<0·05. Given the ecological nature of the disaster and sociocultural, geographical and demographic differences between Bududa district and Kiryandongo district, data were not pooled and the districts were treated independently in the statistical analyses.

## Results

A total of 1078 interviewed participants were analysed among the 1200 people who were eligible in the two districts; sixty-seven households were not available on three visits, thirty-five declined to participate, one was too ill to participate, while nineteen incomplete entries were excluded from the analysis. In Bududa district, the 555 entries that were analysed constituted a participation rate of 93 % for both the affected (*n* 285) and control households (*n* 270) combined. In Kiryandongo district, a participation rate of 87 % was registered from the 523 entries of the affected (*n* 260) and controls (*n* 263) combined.

### Sociodemographic characteristics of the study population

The heads of the household among the controls in Bududa district had a higher mean (sd) age of 43·6 (16·0) years compared with their counterparts in the affected group who were 38·9 (17·0) years old (*P*<0·01). In Kiryandongo district, the household heads of the affected group were on average older with a mean (sd) age of 40·0 (11·9) years compared with the control counterparts who were 37·6 (14·0) years old (*P*=0·04). Differences in household size were significant only in Bududa district, with controls having a higher mean (sd) size compared with the affected households: 6·4 (3·0) *v.* 5·0 (3·2; *P*<0·01).

Despite the difference in education level among affected and control heads of the household in both districts (*P*<0·01), education levels were generally low. The majority of respondents had attained only primary education in both Bududa (64 %) and Kiryandongo districts (71 %). Whereas it was apparent that a majority of households in Bududa (80 %) and Kiryandongo districts (57 %) did not own assets such as commercial land, machines, poultry or livestock to complement their food source, differences between the affected and control households were observed in both districts (*P*<0·01). In addition, despite a difference in the number of households who reported having received relief food in the last 3 years in both districts (*P*<0·01), a larger proportion of affected households in Kiryandongo district (93 %) had received it.

### Variations in household food variety

As shown in Table 1, the performance of households on the number of food varieties consumed (FVS) in each of the twelve food groups that were constituted from the seventy-two food items was generally low in the affected and control households in both districts. In the vegetables group, not more than three food varieties out of the thirteen listed items had been eaten in the 7 d recall period. In addition, food groups which are sources of high-biological-value proteins, such as poultry and eggs, meat, milk and fish, also scored poorly with a mean of less than one variety consumed in the two districts.

**Table 1:** Food variety scores among households affected by the 2010 landslide disaster and control (unaffected) households in the two districts, Uganda, 19 November 2012–21 December 2012

		Bududa district (*n* 555)					Kiryandongo district (*n* 523)				
	No. of food	Affected (*n* 285)		Control (*n* 270)			Affected (*n* 260)		Control (*n* 263)		
Food group	items (*n* 72)	Mean	sd	Mean	sd	*P*	Mean	sd	Mean	sd	*P*
Cereals and grains	5	1·0	0·5	0·6	0·6	<0·01	1·3	0·8	1·3	0·9	0·54
Starchy roots and tubers	7	0·9	0·9	0·6	0·8	<0·01	1·5	1·2	1·2	1·0	0·01
Legumes and pulses	5	0·8	0·6	0·9	0·6	0·01	1·0	0·7	1·1	0·9	0·10
Vegetables	13	2·6	1·3	2·1	1·5	<0·01	2·0	1·8	2·1	1·6	0·45
Fruits and fruit juices	12	1·3	1·1	1·0	1·3	0·01	0·7	1·1	1·1	1·3	<0·01
Poultry and eggs	4	0·2	0·4	0·1	0·3	<0·01	0·4	0·6	0·1	0·4	<0·01
Meat and meat products	6	0·2	0·4	0·2	0·5	0·29	0·2	0·5	0·3	0·6	0·55
Milk and milk products	6	0·5	0·5	0·4	0·5	<0·01	0·4	0·5	0·2	0·4	<0·01
Fish and fish products	3	0·0	0·1	0·0	0·2	0·27	0·0	0·2	0·1	0·3	<0·01
Fats and oils	3	0·8	0·4	0·7	0·6	0·03	0·8	0·7	0·6	0·6	<0·01
Refined sugar and confectioneries	3	0·7	0·5	0·6	0·6	<0·01	0·6	0·5	0·5	0·5	0·01
Carbonated beverages, spices and condiments	5	2·0	0·7	1·8	0·9	0·03	1·7	0·8	1·7	1·1	0·79
Total food variety score		10·8	3·8	8·9	4·7	<0·01	10·4	6·4	10·2	5·6	0·55

In Bududa district, the disaster-affected households scored significantly higher FVS than their control counterparts in nine out of the twelve food groups: cereals and grains; starchy roots and tubers; vegetables; fruits and fruit juices; poultry and eggs; milk and milk products; fats and oils; refined sugar and confectioneries; and carbonated beverages, spices and condiments. On the other hand, the control households in the district scored higher FVS than their affected counterparts on the legumes and pulses group only. In Kiryandongo district, the disaster-affected households scored higher FVS than their control counterparts in five out of the twelve food groups: starchy roots and tubers; poultry and eggs; milk and milk products; fats and oils; and refined sugar and confectioneries. On the other hand, control households in the district scored higher on fruits and fruit juices, and fish and fish products. Overall, the affected households in Bududa district scored a higher total crude mean (sd) of FVS than the controls: 10·8 (3·8) *v.* 8·9 (4·7; *P*<0·01). The corresponding scores in Kiryandongo district were not significantly different (Table 1).

On further stratification of crude FVS by sociodemographic characteristics (Table 2), higher FVS among affected than control households (*P*<0·05) were sustained in Bududa district when we considered: males; females; education less than primary school; households whose main source of livelihood was either farming or otherwise; not having assets to complement food source; and not having received relief food in the preceding 3 years. The only variable in Bududa district where the controls scored higher on food varieties than the affected counterparts was on households who had received relief food in the preceding 3 years. In Kiryandongo district, the only statistical difference was observed in non-recipients of relief food in the preceding 3 years: the controls scored higher than the affected group (*P*<0·01).

**Table 2:** Crude differences in food variety scores and severity of household food insecurity coping strategies to food insecurity among households affected by the 2010 landslide disaster and control (unaffected) households in each district, Uganda, 19 November 2012–21 December 2012

	Bududa district (*n* 555)						Kiryandongo district (*n* 523)					
		Affected (*n* 285)		Controls (*n* 270)				Affected (*n* 260)		Controls (*n* 263)		
	*n*	Mean	sd	Mean	sd	*P*	*n*	Mean	sd	Mean	sd	*P*
(a) Household food variety scores												
Gender of the interviewed head of the household												
Male	373	10·5	3·7	9·4	4·7	0·02	273	11·3	6·8	10·0	5·7	0·10
Female	182	11·4	3·9	8·0	4·5	<0·01	250	9·7	5·9	10·3	5·5	0·39
Education level of the head of the household												
Primary school and less	481	10·8	3·7	8·9	4·5	<0·01	452	9·9	5·9	9·9	5·5	0·95
Beyond primary school	74	11·6	4·7	9·4	5·5	0·12	71	13·5	8·0	12·3	6·2	0·50
Main source of livelihood												
Farming	500	10·8	3·7	9·3	4·7	<0·01	448	10·4	6·4	9·9	5·2	0·37
Others	55	11·4	5·5	7·4	4·5	0·01	75	10·9	6·4	11·5	7·4	0·69
Existence of assets that complement food source												
Yes	110	11·0	4·0	10·5	4·1	0·63	227	11·8	6·5	10·5	5·8	0·10
No	445	10·7	3·7	8·9	4·7	<0·01	296	8·8	5·8	10·0	5·6	0·08
Received relief food in the last 3 years preceding the interview												
Yes	92	10·8	4·3	13·1	5·6	0·04	246	10·8	6·4	11·3	5·1	0·89
No	463	10·8	3·6	8·5	4·4	<0·01	277	6·0	3·5	10·1	5·6	<0·01
(b) Severity of household coping strategies												
Gender of the interviewed head of the household												
Male	373	19·2	19·0	27·0	20·7	<0·01	273	22·6	25·8	11·9	13·7	<0·01
Female	182	18·5	16·3	32·9	23·5	<0·01	250	20·8	27·7	10·4	13·8	<0·01
Education level of the head of the household												
Primary school and less	481	18·8	17·9	29·6	22·2	<0·01	452	21·4	27·5	11·1	14·0	<0·01
Beyond primary school	74	21·5	20·7	26·0	19·5	0·38	71	23·4	22·8	12·5	11·9	0·02
Main source of livelihood												
Farming	500	19·1	17·8	29·7	21·7	<0·01	448	20·5	25·4	11·3	14·0	<0·01
Others	55	16·8	23·7	24·3	21·6	0·28	75	29·1	33·8	10·9	12·7	<0·01
Existence of assets that complement food source												
Yes	110	19·8	20·1	38·3	28·1	<0·01	227	21·0	25·4	11·6	15·7	<0·01
No	445	18·6	17·1	28·2	21·2	<0·01	296	22·5	28·5	11·1	12·8	<0·01
Received relief food in the last 3 years preceding the interview												
Yes	92	20·0	16·8	42·1	25·0	<0·01	246	20·1	25·6	16·0	13·7	0·75
No	463	18·7	18·5	27·4	20·9	<0·01	277	42·8	34·1	11·2	13·8	<0·01

On adjusting for the sociodemographic covariates (Table 3), the multivariate analysis model showed that in Bududa district the affected households exhibited higher mean (se) scores of FVS compared with controls: 10·7 (0·3) *v.* 9·1 (0·3; *P*<0·01). Households whose main source of livelihood was farming also had a higher mean (se) FVS than their counterparts with other livelihoods: 10·1 (0·2) *v.* 8·7 (0·6; *P*=0·02). Additionally, recipients of relief food in the district also had higher mean (se) FVS than non-recipients when the disaster and covariates were controlled: 11·1 (0·5) *v.* 9·7 (0·2; *P*=0·01). On the contrary, in Kiryandongo the control households scored higher mean (se) FVS than affected households when sociodemographic covariates were controlled: 12·2 (0·7) *v.* 8·4 (0·7; *P*<0·01). In addition, higher adjusted mean (se) FVS in the district were observed with: education above primary school compared with primary school and less, 12·6 (0·7) *v.* 9·9 (0·3; *P*<0·01); having owned relevant assets that complemented food source compared with those without, 11·2 (0·4) *v.* 9·6 (0·3; *P*<0·01); and recipients of relief food compared with non-recipients, 12·3 (0·7) *v.* 8·5 (0·7; *P*<0·01).

**Table 3:** Adjusted differences in household food variety and severity of food insecurity coping strategies in the two districts, Uganda, 19 November 2012–21 December 2012

	Bududa district								Kiryandongo district*							
		ANCOVA†								ANCOVA†						
		Food variety‡			Severity of coping§			MANCOVA||		Food variety‡			Severity of coping§			MANCOVA||
Variable	*n*	Mean	se	*P*	Mean	se	*P*	*P*	*n*	Mean	se	*P*	Mean	SE	*P*	*P*
Disaster																
Affected	285	10·7	0·3	<0·01	19·2	1·2	<0·01	<0·01	259	8·4	0·7	<0·01	30·0	2·5	<0·01	<0·01
Controls	270	9·1	0·3		28·6	1·3			259	12·2	0·7		3·0	2·5		
Gender																
Male	373	10·0	0·2	0·66	23·2	1·0	0·31	0·53	270	10·6	0·4	0·26	17·2	1·3	0·43	0·39
Female	182	9·8	0·3		25·0	1·4			248	10·0	0·4		15·7	1·4		
Education																
Above primary school	74	10·5	0·5	0·21	22·8	2·3	0·64	0·40	71	12·6	0·7	<0·01	17·9	2·6	0·56	<0·01
Primary and less	481	9·8	0·2		23·9	0·9			447	9·9	0·3		16·3	1·0		
Main livelihood																
Farming	500	10·1	0·2	0·02	24·2	0·9	0·13	0·03	443	10·2	0·3	0·46	15·9	1·0	0·15	0·27
Others	55	8·7	0·6		20·0	2·7			75	10·8	0·7		19·8	2·5		
Had assets to complement food source																
Yes	110	10·1	0·4	0·63	24·5	2·0	0·71	0·84	225	11·2	0·4	<0·01	15·9	1·4	0·59	0·01
No	445	9·9	0·2		23·6	0·9			293	9·6	0·3		16·9	1·3		
Having received relief food																
Yes	92	11·1	0·5	0·01	27·2	2·1	0·07	0·01	245	12·3	0·7	<0·01	7·2	2·6	<0·01	<0·01
No	463	9·7	0·2		23·1	0·9			273	8·5	0·7		24·8	2·4		

* There are five missing values for age in the district: four in the controls and one in the affected group.

† Test for univariate effect of each variable on the outcome after adjusting for covariates.

‡ Covariates in the model included whether a household was affected by the disaster, head of the household’s gender, age, education attained, household size, main source of livelihood, existence of assets to complement food source, whether the household had received relief food and severity of household food insecurity coping scores.

§ Covariates in the model included whether a household was affected by the disaster, head of the household’s gender, age, education attained, household size, main source of livelihood, existence of assets to complement food source, whether the household had received relief food and food variety scores.

|| Test for multivariate effect of each variable on both outcomes after adjusting for covariates. Given two dependent variables in the model, Hotelling’s Trace value is reported.

### Variations in household food insecurity coping strategies

Table 4 shows the reported number of times the households had adopted each of the eleven food insecurity coping strategies and the assigned weights of severity generated from the pre-test. In Bududa district, the affected households experienced significantly higher coping frequencies compared with control counterparts on two of the eleven coping strategies: purchasing of food on credit and seeking food assistance from neighbours, friends and relatives. On the other hand, the controls in the district experienced significantly higher coping frequencies compared with the affected group on four strategies: relying on less preferred and less expensive food; limiting portion sizes at meal time; parents eating less due to there not being enough food; and children going to bed hungry due to there not being enough food to eat. In Kiryandongo district, the affected households experienced significantly higher frequencies compared with controls on six strategies: relying on less preferred and less expensive foods; seeking financial credit to buy food; seeking food assistance from neighbours, friends and relatives; children going to bed hungry due to there not being enough food to eat; children being allowed to roam and eat elsewhere due to there not being enough food at home; and skipping a day without a household meal due to there not being enough food.

**Table 4:** Frequency of household food insecurity coping strategies among households affected by the 2010 landslide disaster and control (unaffected) households in the two districts, Uganda, 19 November 2012–21 December 2012

		Bududa district (*n* 555)					Kiryandongo district (*n* 523)				
	Severity	Affected (*n* 285)		Controls (*n* 270)			Affected (*n* 260)		Control (*n* 263)		
Coping strategy	weight	Mean	sd	Mean	sd	*P*	Mean	sd	Mean	sd	*P*
Rely on less preferred and less expensive food	1	2·3	2·2	3·3	2·1	<0·01	3·1	2·0	1·9	1·8	<0·01
Limit portion sizes at meals	2	2·6	2·4	3·1	2·1	0·03	2·4	1·4	2·3	2·4	0·91
Reduce food for adults so children can eat	2	3·0	2·3	2·5	1·9	0·08	2·7	1·5	2·7	2·2	0·99
Parents eat less because there is not enough food	2	2·7	2·5	3·7	2·5	<0·01	2·9	1·5	3·1	2·7	0·54
Purchase food on credit	2	2·6	1·9	2·1	1·6	<0·01	1·7	1·3	1·6	1·6	0·57
Seek financial credit to buy food	3	1·6	1·4	1·6	1·7	0·96	2·2	1·4	1·2	1·1	<0·01
Children eat less food due to there not being enough	3	2·7	1·9	2·2	1·9	0·06	2·6	1·5	2·2	1·7	0·17
Seek food assistance from neighbours, friends and relatives	3	1·6	1·8	1·1	1·2	<0·05	2·1	1·5	1·2	1·7	<0·01
Children go to bed hungry because there is not enough food to eat	4	1·2	1·3	1·6	1·5	<0·05	2·3	1·6	0·9	0·8	<0·01
Children allowed to roam and eat elsewhere	4	2·7	2·4	1·6	1·6	0·16	2·8	1·8	1·4	1·4	<0·01
Skip a day without eating a household meal	4	1·3	1·1	1·2	0·9	0·43	2·1	1·3	1·7	1·1	0·01
Total weighted coping strategy score		19·0	18·1	28·9	21·7	<0·01	21·7	26·8	11·3	13·8	<0·01

Values are scores.

Overall, the severity of household food insecurity coping strategies based on crude weighted scores showed that the control households in Bududa district exhibited higher mean (sd) coping scores than their affected counterparts: 28·9 (21·7) *v.* 19·0 (18·1; *P*<0·01). However, the opposite was observed in Kiryandongo district as affected households had higher mean (sd) scores than their control counterparts: 21·7 (26·8) *v.* 11·3 (13·8; *P*<0·01; Table 4).

On further stratification by sociodemographic variables (Table 2), the trends in both districts remained consistent with a few exceptions. In Bududa district, crude scores on severity of coping were significantly higher for controls than affected counterparts among: both genders of household heads; those whose education was primary and below; those whose main source of livelihood was farming; those with or without assets to complement food source; as well as those who had either received or never received relief food. In Kiryandongo district, the trends were opposite to Bududa: crude differences in severity of coping to food insecurity were higher in affected households than their control counterparts with the exception of households who reported having received relief food in the 3 years prior to the interview.

Table 5 shows the likelihood to adopt each of the eleven coping strategies among the affected and controls. In Bududa district, the affected households were more than two times more likely to skip a day without eating a household meal (breakfast, lunch or supper) compared with their control counterparts. However, it was less likely for affected households in the district to: rely on less preferred and less expensive food; limit portion sizes at meals; sanction parents to eat less; seek credit to buy food; let children eat less due to there not being enough food; seek food assistance from neighbours, friends and relatives; and allow children to go to bed hungry due to there not being enough food.

**Table 5:** The likelihood of households affected by the 2010 landslide disaster and control (unaffected) households in each district to adopt each food insecurity coping strategy when food is insufficient, Uganda, 19 November 2012–21 December 2012

	Bududa district (*n* 555)							Kiryandongo district (*n* 523)						
Coping strategy	*n*	%	Affected	Controls	OR	95 % CI	*P*	*n*	%	Affected	Controls	OR	95 % CI	*P*
Rely on less preferred and less expensive food														
Yes	341	61·4	117	224	0·14	0·10, 0·21	<0·01	208	39·8	130	78	2·37	1·66, 3·40	<0·01
No	214	38·6	168	46				315	60·2	130	185			
Limit portion sizes at meals														
Yes	381	68·6	170	211	0·41	0·29, 0·60	<0·01	226	43·2	135	91	2·04	1·44, 2·90	<0·01
No	174	31·4	115	59				297	56·8	125	172			
Reduce food for adults so children can eat														
Yes	219	39·5	113	106	1·02	0·72, 1·43	0·93	230	44·0	134	96	1·85	1·30, 2·62	<0·01
No	336	60·5	172	162				293	56·0	126	167			
Parents eat less because there is not enough food														
Yes	314	56·6	128	186	0·37	0·26, 0·52	<0·01	181	34·6	106	75	1·73	1·20, 2·48	<0·01
No	241	43·4	157	84				342	65·4	154	188			
Purchase food on credit														
Yes	430	77·5	216	214	0·82	0·55, 1·22	0·36	215	41·1	113	102	1·21	0·86, 1·72	0·29
No	125	22·5	69	56				308	58·9	147	161			
Seek financial credit to buy food														
Yes	224	40·4	65	159	0·21	0·14, 0·30	<0·01	143	27·3	70	73	0·96	0·65, 1·41	0·85
No	331	59·6	220	111				380	72·7	190	190			
Children eat less food due to there not being enough														
Yes	309	55·7	124	185	0·35	0·25, 0·50	<0·01	164	31·4	97	67	1·74	1·20, 2·53	0·01
No	246	44·3	161	85				359	68·6	163	196			
Seek food assistance from neighbours, friends and relatives														
Yes	198	35·7	76	122	0·44	0·31, 0·63	<0·01	173	33·1	104	69	1·87	1·29, 2·71	<0·01
No	357	64·3	209	148				350	66·9	156	194			
Children go to bed hungry because there is not enough food to eat														
Yes	149	26·8	60	89	0·54	0·37, 0·79	<0·01	111	21·2	57	54	1·09	0·72, 1·65	0·75
No	406	73·2	225	181				412	78·8	203	209			
Children allowed to roam and eat elsewhere														
Yes	35	6·3	13	22	0·54	0·27, 1·09	0·12	60	11·5	31	29	1·09	0·64, 1·87	0·79
No	520	93·7	272	248				463	88·5	229	234			
Skip a day without eating a household meal														
Yes	202	36·4	130	72	2·31	1·62, 3·29	<0·01	174	33·3	103	71	1·77	1·23, 2·57	<0·01
No	353	63·6	155	198				349	66·7	157	192			

In Kiryandongo district the affected households were more than two times more likely than their control counterparts to rely on less preferred and less expensive food, and to limit portion sizes at meals. Moreover, the affected households also had about a twice higher likelihood than controls to: reduce food for adults so children could eat; sanction parents to eat less due to there not being enough food; allow children eat less food due to there not being enough; seek food assistance from neighbours, relatives and friends; and skip a day without eating a household meal.

On adjusting for the sociodemographic covariates (Table 3), the multivariate analysis model showed that in Bududa district the control households still exhibited higher mean (se) scores of severity of coping compared with affected counterparts. Previous crude differences among affected and controls in the district were extinguished when the covariates were controlled. In Kiryandongo district high severity scores were observed among affected households compared with control counterparts, and among those households who did not receive relief food compared with those who received it, when covariates were controlled.

### Multivariate effects on both food variety scores and severity of coping to food insecurity

Given the positive correlation between the two dependent variables, namely food variety and coping strategies, in the multivariate model, the MANCOVA test of multivariate effect showed that the disaster predicted both outcomes when sociodemographic variables were controlled in Bududa and Kiryandongo districts (*P*<0·01 in both; Table 3). The model also showed that having received relief predicted both outcomes when sociodemographic variables were controlled in Bududa (*P*=0·01) and Kiryandongo districts (*P*<0·01). Distinctively, the main source of livelihood predicted both outcomes in Bududa district only (*P*=0·03), while in Kiryandongo district both outcomes could be predicted by education (*P*<0·01) and owning relevant assets that complemented food source (*P*=0·01).

## Discussion

Our main findings showed opposite trends of results in both districts. In Bududa district, affected households had consumed more food varieties than controls, but in Kiryandongo it was the controls who consumed more varieties. In addition, in Bududa district the control households experienced higher scores of severe coping to food insecurity, but in Kiryandongo it was the affected households who exhibited high scores. Generally, surveyed households in the two districts consumed diets with low food varieties as most had used fewer than eleven out of the seventy-two commonly eaten food varieties that had been listed in twelve food variety groups. Sources of high-biological-value protein such as fish, meat, poultry, eggs and milk varieties also scored poorly in both districts. However, we observed that households who had ever received relief food consumed more varieties than those who did not receive it when other factors were taken into account in both districts. Additionally, affected households in both districts had a two times greater likelihood than controls to skip a day without eating a household meal, while seeking food assistance from neighbours, friends and relatives was also a significant practice of affected households in both districts.

Consistent with our previous findings on household food insecurity and diet diversity after the landslide^(^ ^21^ ^)^, food variety and coping strategies also exhibited a positive correlation and the multivariate model showed that being affected by the disaster and having received relief predicted both outcomes when sociodemographic variables were controlled in both districts. Distinctively, the main source of livelihood could predict both outcomes in Bududa district only, while in Kiryandongo both outcomes could be predicted by education and owning relevant assets that complement food source.

The indication that the disaster-affected households had higher FVS in Bududa district, against a backdrop of most affected households having received relief assistance and the higher FVS among recipients of relief food in both districts, is consistent with findings from previous studies showing that relief food often improves access to diverse food^(^ ^38^ ^,^ ^39^ ^)^. This seems to imply that relief food provision positively influenced food varieties consumed by the households. However, there is need for caution in interpreting these findings especially where relief food is not exclusively controlled from being accessed by other non-targeted beneficiaries in the neighbouring communities being compared. Moreover, as seen in the present case, the control households in Bududa district who had received relief food scored higher on food variety scores than their counterparts in the affected group.

Tracing the actual quantity and quality of relief food supplied, the logistics involved and actual utilization by households is vital in assessing the impact of emergency food and nutrition, and for accountability, during disaster management. Whereas the Government reports did indicate that relief food in the form of the common staples of maize/corn flour (*Zea mays*) and beans (*Phaseolus vulgaris*) was procured and supplied to the landslide victims^(^ ^18^ ^)^, the situation was complicated when the Uganda Human Rights Commission report indicated that truckloads of distributed relief food were being transported with impunity out of the resettlement area to the markets in Kiryandongo district where the landslide victims had been resettled^(^ ^40^ ^)^. This implies a potential challenge to the assessment and monitoring of adequacy of relief food.

The low scores on high-biological-value protein such as fish, meat, poultry, eggs and milk implies a possible difficulty faced by most households in ensuing the availability and accessibility to these foods in their diet. Moreover, animal-source protein foods are often expensive and therefore accessibility is low in many parts of Africa where income levels are generally low^(^ ^36^ ^)^. In Uganda, Kikafunda and Tumwine^(^ ^41^ ^)^ established that incomes and occupation influence access to animal-source foods and child nutritional status. In some other cases, low levels of education and awareness on optimal nutrition practices have also contributed to poor diet diversity in Uganda^(^ ^41^ ^,^ ^42^ ^)^. We observed a similar trend in the present study as household heads with an education beyond primary school scored consistently higher FVS than their counterparts with a low education in the affected and control groups of both districts.

Despite the absence of a national food and diet guide that would specify the normal Ugandan diet from which comparisons and variations would be assessed, the low scores on cereals and grains, starchy roots and tubers, and legumes and pulses seemed unusual since they are widely accessible and consumed in many parts of the country^(^ ^43^ ^,^ ^44^ ^)^. Moreover, a recent national population and housing census indicated that on average more than half of the population (51 %) had reported eating two meals per day, about 35 % had three meals and 12 % had one meal per day^(^ ^45^ ^)^; implying that suboptimal meal patterns were widespread in the country.

There was no consistent link between severities of coping strategies and being a disaster victim. Higher scores for severe coping strategies were observed among the controls in Bududa district, but in Kiryandongo district this was observed in the affected group. However, seeking food assistance from neighbours, family and friends was a consistent strategy deployed by disaster-affected households in both districts, while coping was exacerbated by a lack of assets to complement food source. This corroborates other findings that link household sociodemographic characteristics including assets deficiency to increased economic strains on the household^(^ ^46^ ^)^.

The potential lack of community safety nets, and a possible deficiency of social and administrative structures of the Government as described in our publications elsewhere^(^ ^19^ ^,^ ^47^ ^)^, may explain why affected households in both districts preferred to seek food assistance from neighbours, friends and relatives. No wonder the risk of skipping an entire day without eating a household meal was two times more likely among the affected households. Although community, family and neighbourhood safety nets are still a viable alternative in the Ugandan context, the capital base of supportive families, friends and close relatives is often small and may not provide long-term prospects for achieving and maintaining adequate food in the household. Government-instituted structures to deal with social security safety nets are necessary as part of the disaster preparedness and management framework, and can help to check poor FVS and severe coping strategies at household level.

As reported in our other recent findings from the same study populations^(^ ^20^ ^,^ ^21^ ^)^, our design did not correct for possible effects of seasonal variations on food choice, food variety and coping strategies. Other limitations were the lack of measures of body composition and biomarkers for nutritional intake, and possible recall bias. Although we targeted the available head of the household with preference to women, the predominantly male-headed households involved in the study might have also limited scope of information on food used by the household given the vital role played by women in food and nutrition security at the household level^(^ ^4^ ^,^ ^48^ ^–^ ^50^ ^)^. Furthermore, the ecological nature of the disaster also prevented sampling of both affected and control households from a homogeneous population; the disaster was widespread in social and geographical scope, and the subsequent resettlement was into a specific and previously unoccupied distant locality in Kiryandongo district. It was therefore difficult to locate suitable affected and controls households from within the same homogeneous population group. Moreover, due to ecological, social and demographic differences between the two districts, we decided to limit our comparisons between the affected and control households within each district rather than across the two districts. Given the inconsistencies in scores between the two districts, to the extent that the directions of some results are opposite, generalizability of findings could have been undermined beyond geographical settings and types of natural disaster.

The major strength of our study was in the adaptation of a detailed semi-quantitative FFQ that listed individual food items under twelve food groups so as to score both diet diversity scores^(^ ^21^ ^)^ and FVS. We also adapted validated household food access experiences, child hunger and food insecurity coping strategies from complementary tools that have been used for assessing food insecurity in resource-limited settings, i.e. the HFIAS^(^ ^32^ ^)^, the CCHIP index^(^ ^33^ ^,^ ^51^ ^)^ and the CSI ^(^ ^6^ ^,^ ^7^ ^)^, respectively.

## Conclusion

In conclusion, the present study has shown that intake of food varieties and associated coping strategies of households varied in both groups in the two districts with modest implications of disaster exposure. Differences in FVS were opposite in both districts as they seemed to favour the affected households in Bududa district and control households in Kiryandongo district. Despite higher FVS among households who had received relief food in both districts, the opposite results on FVS among affected households in Bududa and Kiryandongo may not be sufficiently explained by humanitarian relief food that was made available and accessible to the disaster victims. Moreover, controls who had also received relief food in Bududa district reported relatively higher variety scores than affected counterparts. In addition, the low use of food varieties rich in high-biological-value protein in the surveyed districts is seemingly a concern that has implications on the system responsible for disaster relief operations.

Given the relatively higher likelihood to skip meals in the disaster-affected households, urgency in delivery of relief response should be a central aspect in disaster management operations. There seems to be a lack of robust community safety nets and a possible deficiency of public social administrative structures of the Government. Going forward, such a situation calls for strengthening a decentralized approach to disaster management operations so as to accommodate and implement tailor-made disaster-specific assessments and interventions that address the unique challenges in different geographical areas.

Whereas emergency nutrition recommendations by the UN agencies have specified a minimum target of 8786 kJ (2100 kcal) during emergency situations^(^ ^52^ ^)^, translating this recommendation into viable food variety recommendations and food exchange lists is still vital yet unaccomplished in many countries. Moreover, the situation becomes more complex when country-level emergency response programmes lack specific food-based guidelines and minimum relief food specifications for emergency interventions. Essentially, an individual-specific food and nutrition package for disaster victims needs to be clearly defined by policy and legislation in Uganda and other parts of the world so as to guide institutional actions during response. Such a package could take into account the dimension of adequate food within the wider context of the primary obligations of the State to respect, protect and fulfil the human right to adequate food of vulnerable rights holders. In effect, a human rights-based emergency response approach that is based on country-specific relief food guidelines and specifications may have potential to offset the undesirable food insecurity coping strategies that can lead a household into precarious situations of hunger, starvation and related consequences in the aftermath of disasters.
